# Miscellany

**Published:** 1895-09

**Authors:** 


### Miscellany.

### CARE OF PERSONS FOUND UNCONSCIOUS ON THE STREETS.

THE Medical Society of the County of Kings has issued a circular concerning the care of persons found unconscious on the streets, which contains the following judicious recommendations. It is the hope of the committee making the report that all concerned will coöperate in such a manner as to insure something like concerted action. If this is done, then persons who are rendered unconscious from any cause on the streets or elsewhere, will receive prompt medical and humane treatment, and will escape the danger of being thrust in a cell as "drunks," and there left to sleep off the supposed debauch, which in no inconsiderable number of cases has proved to be "a sleep that knows no waking."

RECOMMENDATIONS.

1. Whenever a person is found in an unconscious or semi-conscious state on the street or elsewhere, away from his own home, the police, when notified of such case, shall immediately summon medical aid ; sending for the ambulance surgeon, or for the police surgeon ; or in towns, where there are no such officials, then for the nearest physician, who should be compensated for his services by the authorities.

2. The police shall not decide as to the disposition of such a case, but must await the decision of the ambulance surgeon, police surgeon, or of the physician called and must act in accordance with such decision.

3. A police officer who acts in opposition to such decision should be by the ambulance surgeon, police surgeon, or the physician, reported to the police commissioner, who should subject such officer to discipline, rules governing such cases having previously been made and promulgated.

4. Ambulance surgeons should give prompt and immediate aid to patients found in the condition hitherto described and remove them to the nearest hospital, or to their homes when ascertainable, according as their judgment dictates is the best course to pursue in the interest of the patients. The existence of an alcoholic complication in the case should in nowise adversely influence the surgeon or physician called as to the disposition of the case, as such a complication often renders skilful medical treatment the more imperative.

5. Ambulance surgeons and other medical men brought in contact with cases in which alcoholism is a frequent complication, should be reminded that this condition often renders an immediate diagnosis impossible in the most serious and oftentimes fatal forms of cerebral disease and injury, as well as in other diseased conditions.

6. The examination of ambulance surgeons should include the differential diagnosis of alcoholic coma from other forms of coma, and the various diseases or injuries that may produce a condition simulating alcoholic intoxication.

7. Hospital authorities receiving financial aid from the city should not refuse admittance to patients suffering from supposed alcoholism, for in so doing they are liable to be contributory to the death of such patients. They should know that if the condition be one of uncomplicated alcoholism, this fact will in a short time be revealed, and other disposition may be subsequently made of the case ; while, if the patient is so affected as to need immediate and skilful treatment, his rejection by the hospital authorities may conduce to a fatal result. If they refuse to receive such cases, because complicated with alcoholism, they should be held legally responsible for the results. And, further, if such refusal is persistent after their attention has been called to the matter, the city authorities should strike the name of such hospital from its list of beneficiaries.

8. The municipal authorities should also consider the question of the establishment of a special emergency hospital, or hospitals, conveniently located with reference to the various districts of the city ; or a system, similar to that of the Bureau d'Admission in Paris, connected with which there is a special hospital for all cases of alcoholism, or cases complicated with alcoholism, that may occur in the streets of that city. Or the authorities might consider the establishment of a special department in connection with the hospitals of the city, similar to the "alcoholic wards" of Bellevue Hospital, New York, where more than 4,000 alcoholics are annually treated. Such a plan would relieve the general hospitals of the burden of such cases, or compel them to make special provision for their care. Should the existing methods prove inadequate, the committee recommends some such plan as is here outlined.

---

Mr. J. H. Ullenbruch, manufacturing optician, 274 Main street, Buffalo, has received a large stock of the new combination sterilising thermometer. This instrument ought to be in the hands of every family that has small children. It is especially useful in sterilising milk, which should always be done with bottle-fed children. Mr. Ullenbruch also carries a complete line of the best clinical thermometers, an instrument that is always an important part of a physician's armamentarium.

---

The King thermometer syringe, illustrated in the July issue of the Journal, has been placed on the market at the net price of $2.00 instead of $2.25 as originally stated. This brings it down to about as low a price as other fountain syringes of the same size and quality and gives the additional advantage of a thermometer attachment.

